# Comparison of ultrasound-assisted and pure fluoroscopy-guided extracorporeal shockwave lithotripsy for renal stones

**DOI:** 10.1186/s12894-020-00756-6

**Published:** 2020-11-10

**Authors:** Tsung-Hsin Chang, Wun-Rong Lin, Wei-Kung Tsai, Pai-Kai Chiang, Marcelo Chen, Jen-Shu Tseng, Allen W. Chiu

**Affiliations:** 1 Department of Urology, MacKay Memorial Hospital, No. 92, Sec. 2, Zhongshan N. Rd., Taipei City, 10449 Taiwan; 2 Mackay Medical College, No.46, Sec. 3, Zhongzheng Rd., Sanzhi Dist., New Taipei City, 252 Taiwan; 3 Mackay Junior College of Medicine, Beitou District, Nursing, and Management, No.92, Shengjing Road, Taipei City, 11272 Taiwan; 4 School of Medicine, National Yang-Ming University, No.145, Zhengzhou Rd., Datong Dist., Taipei City, 10341 Taiwan

**Keywords:** Shock wave lithotripsy, Nephrolithiasis, Kidney, Ultrasonography

## Abstract

**Background:**

In this study, we aimed to compare the efficacy and clinical outcomes of shock wave lithotripsy (SWL) for patients with renal stones using pure fluoroscopy (FS) or ultrasound-assisted (USa) localization with two lithotripters.

**Methods:**

We retrospectively identified 425 patients with renal calculi who underwent SWL with either a LiteMed LM-9200 ELMA lithotripter (209 cases), which combined ultrasound and fluoroscopic stone targeting or a Medispec EM-1000 lithotripter machine (216 cases), which used fluoroscopy for stone localization and tracking. The patient demographic data, stone-free rates, stone disintegration rates, retreatment rates and complication rates were analyzed.

**Results:**

The USa group had a significantly higher overall stone-free rate (43.6 vs. 28.2%, p < 0.001) and stone disintegration rate (85.6 vs. 64.3%, p < 0.001), as well as a significantly lower retreatment rate (14.8 vs. 35.6%, p < 0.001) and complication rate (1.9 vs. 5.5%, p = 0.031) compared with the FS group. This superiority remained significant in the stone size < 1 cm stratified group. In the stone size > 1 cm group, the stone-free rate (32.4 vs. 17.8%, p = 0.028), disintegration rate (89.2 vs. 54.8%, p = 0.031) and retreatment rate (21.6 vs. 53.4%, p < 0.001) were still significantly better in the USa group, however there was no significant difference in the complication rate. The most common complication was post-SWL-related flank pain.

**Conclusion:**

SWL is a safe and non-invasive way of treating renal stones. This study compared two electromagnetic shock wave machines with different stone tracking systems. LiteMed LM-9200 ELMA lithotripter, which combined ultrasound and fluoroscopic stone targeting outperformed Medispec EM-1000 lithotripter, which used fluoroscopy for stone localization and tracking, with better stone-free rates and disintegration rates, as well as lower retreatment rates and complications with possible reduced radiation exposure.

## Background

Extracorporeal shock wave lithotripsy (SWL) has been used as a non-invasive method of urolithiasis management since the 1980’s [1]. It has been proven to be a safe, efficient way of treating non-infected upper urinary tract stones and remains one of the most commonly used treatment modality for renal stones worldwide today.

Contemporary studies have shown that the success rate of SWL for renal stones ranges from 47 to 92% [2–7]. This highly variable result is due to various crucial factors, including stone composition and size [8–11], skin to stone distance [12], lithotripter energy power [13] and frequency [14, 15], patient positioning [16], and patient tolerance and respiration [16, 17]. Less than half of the administered shockwaves may be accurately focused on the targeted calculus [18], and the excessive energy and shockwaves can cause damage to the parenchyma and adjacent organs. Chen et al. [19] demonstrated increased accuracy of stone targeting by using an electromagnetic lithotripter integrated with both ultrasound real-time tracking system and traditional fluoroscopy (LiteMed LM-9200 ELMA). Through the alternative use of ultrasound and fluoroscopy, the energy of the lithotripter may be more focused on the target stone during the whole session, thus increasing SWL efficiency. However, only a few studies have compared the efficacy of ultrasound-based lithotripters with fluoroscopy-based lithotripters. It is difficult to compare efficacy between different institutions due to variabilities in treatment protocols and operators. The current study compared the efficacy and clinical outcomes of two electromagnetic lithotripters using different stone localization methods (LiteMed LM-9200 ELMA lithotripter: combined ultrasound and fluoroscopic stone targeting; Medispec EM-1000 lithotripter: pure fluoroscopic stone targeting), in a single center with 2 hospital branches, using the same group of urologists and the same set of patients and surgical indications. The machines were operated by the same two experienced technicians, which minimizes operator and institutional related bias.

The aim of the current study was to compare the efficacy and clinical outcomes of these two different stone localization modalities in the treatment of renal stones in one single medical center.

## Methods

The present study retrospectively reviewed the medical records of patients with radio-opaque kidney stones with stone size between 0.5 to 2.0 cm who underwent SWL in a single medical center with 2 hospital branches between January 2013 and March 2019. Exclusion criteria were patients with congenital anomalies or urinary diversion, pediatric patients or patients who received SWL as a combination therapy with other treatment modalities. Patients with radiolucent stones were also excluded as plain x-rays were used as the standard follow-up modality.

All patients underwent imaging and laboratory studies prior to SWL. Plain abdominal radiography of the kidneys, ureter and bladder (KUB), renal sonography, intravenous urography (IVU) and abdominal computed tomography (CT) were used to diagnose and identify the stones. The size of the stones were measured on the KUB using maximal stone diameters.

Patients underwent SWL at two branches of our medical center with either a LiteMed LM-9200 ELMA lithotripter or Medispec LTD EM-1000 lithotripter, depending on their location. The distance between the two branches of our medical center were about 10 km with about a 20 min driving time and both were located in the northern part of the city. There were no regional characteristics different between the patients of the two branches, such as water composition and eating habits. The energy source of both lithotripters was an electromagnetic shockwave source. The focal length was 145 mm for EM-1000 and 125 mm for LM-9200. All SWL treatments were performed on an ambulatory basis by the same group of urologists. Both machines were operated by the same two experienced technicians. The LiteMed LM-9200 ELMA lithotripter used ultrasonography and fluoroscopy for dual focusing of stone localization and tracking, while the EM1000 machine used only fluoroscopy for stone visualization, with an integrated computerized fluoroscopic X-ray (Visionspec™). The LiteMed LM-9200 machine has a auto-firing system, the shockwaves will only be fired when the stone is in the focal zone.

The same standardized SWL protocol was used for both machines except for the stone localization method. The patient was placed in a supine position without a restraint belt. The cushion size and lubricating jelly used were the same in both branch. and the stone was located by the fluoroscopy in both group. In the USa group, ultrasound was combined use for dual stone localization before initiating the SWL session and during the treatment for real time tracking of the stone. While in the FS group, only pure fluoroscopy was used throughout the whole session. During SWL, the initial energy level was set at 14KV and could gradually increased up to 19 kV if the stones did not show adequate fragmentation under low energy settings. The shock wave rate was set at 60 shocks per minute for both lithotripters. The maximum number of shockwaves delivered in one session was 3,000. Light intravenous propofol and dormicum were used as analgesics or for sedation if the patient suffered from pain or could not tolerate lying still in the supine position throughout the SWL session. The patient’s position was adjusted throughout the SWL session for better stone visualization. If the patient’s stone is still in focal zone, the position of the patient will not be changed to avoid constant de-coupling and re-coupling. In the FS group, the position was checked and adjusted after every 600 shocks or when the patient moved. While in the USa group, positioning was adjusted based on the ultrasound tracking of the stone location throughout the SWL session. All patient received SWL sessions on an ambulatory basis, analgesics with Diclofenac or Acetaminophen were prescribed for post-operative pain control.

Patients were evaluated at 4, 12 and 24 weeks after the SWL session by KUB. Stone-free status was defined as the absence of radiographic residual stones in the follow-up imaging, any residual fragments visible on KUB was considered as non-stone-free. The KUB x-ray. Stone disintegration was defined as the presence of stone destruction, fragmentation or a decrease in size in the post-session KUB compared with the pre-session KUB. Retreatment was defined as the need for further surgical intervention (repeat SWL, rigid ureteroscopic lithotripsy (URSL), retrograde intrarenal surgery (RIRS) or percutaneous nephrolithotomy (PCNL)) for symptomatic patients or patients with residual stone fragments > 0.5 cm. Complications were classified according to the Clavien-Dindo classification system. All data were analyzed and compared using t-tests and chi-squared tests with IBM SPSS statistics version 25.0 software. Statistical significance was set at p < 0.05.

The present study, including its research protocols and data collection, were approved by the Mackay Memorial Hospital Institutional Review Board. All personal information was de-identified prior to data analysis, thus ensuring patient data confidentiality.

## Results

Of the 425 included patients, 209 were treated with USa SWL and 216 were treated with FS SWL. The mean age of patients was 53.3 ± 13.2 years in the USa group and 53.8 ± 12.4 years in the FS group (p = 0.538). Comparison of the two groups revealed that the FS group was significantly more male dominated (p = 0.016) and there were significantly larger stones in the USa group (1.03 ± 0.37 cm vs. 0.87 ± 0.33 cm, p < 0.005) (Table 1).

**Table 1:** Patient demographics and stone characteristics

Characteristic	USa	FS	p value
Number of patients	209	216	
Patient age (mean ± SD)	53.3 ± 13.2	53.8 ± 12.4	0.568
Patient gender			0.016
Male, n (%)	130 (62.2%)	158 (73.1%)	
Female, n (%)	79 (37.8%)	58 (26.8%)	
Stone size (cm)	1.03 ± 0.37	0.87 ± 0.33	0.008
> 1 cm (n)	111	73	
< 1 cm (n)	98	143	

Statistical analysis revealed better treatment outcomes in the USa group. The overall stone free rate was significantly better in the USa group compared with the FS group (USa 43.5% vs. FS 28.2%, p < 0.001), and the overall stone disintegration rates were significantly higher (USa 85.6% vs. FS 64.3%, p < 0.001). Significant lower retreatment (USa 14.8% vs. FS 35.6%, p < 0.001) and complication rates (USa 1.9% vs. FS 5.5%, p = 0.031) were noted in the USa group compared with the FS group (Table 2).

**Table 2:** Overall outcomes, stone free rate, complication, retreatment rate

	US (n = 209)	FS (n = 216)	p value
Stone free rate (%)	91 (43.5)	61 (28.2)	< 0.001
Disintegrate rate (%)	179 (85.6)	139 (64.3)	< 0.001
Retreatment rate (%)	31 (14.8)	77 (35.6)	< 0.001
Complication rate (%)	4 (1.9)	13 (6.0)	0.031

Patients were further stratified by stone size (< 1 cm and ≥ 1 cm), and the results were similar following stratification. In the group with renal stones < 1 cm the USa group had a significantly higher stone free rate (USa 56.1% vs. FS 33.5%, p < 0.001) and stone disintegration rate (USa 81.6% vs. FS 69.2%, p = 0.031) compared with the FS group. The USa group also had a significantly lower retreatment rate (USa 7.1% vs. FS 26.5%, p < 0.001) and complication rate (USa 1.0% vs. FS 6.9%, p = 0.029) (Table 3).

**Table 3:** Stone size < 1 cm, outcomes, stone free rate, complication, retreatment rate

	US (n = 98)	FS (n = 143)	p value
Stone free rate (%)	55 (56.1)	48 (33.5)	< 0.001
Disintegrate rate (%)	80 (81.6)	99 (69.2)	0.031
Retreatment rate (%)	7 (7.1)	38 (26.5)	< 0.001
Complication rate (%)	1 (1.0)	10 (6.9)	0.029

In the group with stones > 1 cm, the USa group still had a significantly better stone free rate (USa 32.4% vs. FS 17.8%, p = 0.028), stone disintegration rate (USa 89.2% vs. FS 54.8%, p < 0.001) and retreatment rate (USa 21.6% vs. FS 53.4%, p < 0.001) compared with the FS group. However, the complication rates were not statistically different between the two groups (US 2.7% vs. FS 4.1%, p = 0.599), but the USa group did still have a lower complication ratio (Table 4).

**Table 4:** Stone size > 1 cm, outcomes, stone free rate, complication, retreatment rate

	US (n = 111)	FS (n = 73)	p value
Stone free rate (%)	36 (32.4)	13/ (17.8)	0.028
Disintegrate rate (%)	99 (89.2)	40 (54.8)	< 0.001
Retreatment rate (%)	24 (21.6)	39 (53.4)	< 0.001
Complication rate (%)	3 (2.7)	3 (4.1)	0.599

The most common complication was post-SWL-related flank pain (Clavien-Dindo grade I), which occurred in 1 (0.5%) USa group patient and 11 (5.1%) FS group patients, however no cases required hospitalization. In all cases symptoms were controlled with intravenous/intramuscular or oral non-steroidal anti-inflammatory drugs (NSAIDs). In the USa group, 1 patient developed ureteral stone street after SWL; the patient had a 1.7 cm renal stone and required further RIRS for obstruction release and residual stone removal (grade 3). Another patient had a urinary tract infection and required hospitalization for intravenous antibiotics (grade 2). In the FS group, 2 patients developed post-SWL ureteral stone street, and one required URSL for stone removal (grade 3); the other patient had stone passage during follow up (grade 2).

## Discussion

SWL has been the primary treatment option for renal and upper urinary calculi since its introduction in the early 1980′s [20]. Two major international urological associations: the European association of urology and the American urological association both recommend SWL as the treatment of choice for small to intermediate renal stones. However, the treatment has certain limitations which may predict poor treatment efficacy, including steep and narrow infundibulum, long lower pole calyx, and shockwave-resistant stones [21, 22].

To maximize the efficacy and outcomes while minimizing complications of SWL, certain options regarding patient and stone characteristics have been proposed [16]. Obese patients and those with a higher body mass index (BMI) may have a larger skin to stone distance revealed during clinical examination, which could lead to unfavorable SWL success rates [12, 23]. Some studies highlighted the importance of anatomical location and the architecture of the stones as measured by CT Hounsfield units [8–11].

Other evidence showed that during SWL sessions, the energy and frequency emitted by the lithotripter notably contributed to stone disintegration. It is recommended that the treatment strategy starts from a low energy level and low frequency rate, and then gradually increases. Starting from a low energy level has been shown to pre-sensitize the kidney and cause renal vasoconstrictions [13, 24], thus reducing renal damage and improving patient tolerance. Low frequency shock wave rates have been proven to be associated with better stone fragmentation in many previous studies [14, 25–27]. Although there is no gold standard for optimal treatment frequency, it has been shown that frequency rates of 60 shocks per minute (1 Hz) and 90 shocks per minute (1.5 Hz) both had better outcomes compared with 120 shocks per minute (2 Hz) [14, 25–27]. Decreasing both the initial energy and shock wave frequency results in a reduced total number of shock waves and energy being delivered, thus reducing the possible damage to the kidney, and therefore decreasing the possibility of complications and improving patient comfort. The patient could be more easily adapted to this treatment, thus minimizing patient movement and the use of sedative agents. In this way, energy delivery could be more focused on the target and stone disruption could be improved. The training and experience of the technicians and operators performing the procedure also has an impact on the success of an SWL session, studies have shown that the stone free rate improves as the operator completes a learning curve [28, 29].

In the current study, the SWL session starts with a low energy (14KV) and low frequency setting (60 Hz) in order to maximize the treatment effect. All of the SWL sessions were performed by the same two technicians who have over 10 years of experience. The authors consider this to be a strength of the current study. Using the same low-frequency, low energy machine setup and having SWL sessions performed by the same two experienced operators, the SWL efficiency could be maximized while minimizing the inter-operator bias and learning curve bias.

Last but not least, the impact of the patient’s stability during the SWL session must be considered. Patient movement and respiration during the SWL session can cause kidney movement [17], thus causing shock waves to be misdirected and focused away from the target. Excessive energy and inaccurate shockwaves may cause damage to the parenchyma and adjacent organs. Even when patients are under sedation or anesthesia during SWL, respiration related mistargeting remains inevitable. Constant tracking of the stone under a continuous fluoroscope could be the solution, however this may cause excessive ionizing radiation exposure. To overcome this problem, Chang et al. developed a real-time tracking ultrasound based system for renal stones [30]. In vitro and animal studies [31] showed the system to be efficient, and it could increase the accuracy of renal stone targeting and the efficiency of stone fragmentation.

The major advantage that ultrasound provides is real-time tracking of the stone without excessive radiation exposure to the patient. Although the dose of radiation during one SWL session may not be much and is not a worrisome dose compared to other invasive radiological procedures, the cumulative effect of ionizing radiation that needs to take into account includes the pre-treatment diagnostic imaging studies, such as CT or IVU, and possible repeat SWL treatment at a later date.

Chen et al. [19] reported on their clinical experience of renal stone treatment using an electromagnetic lithotripter integrated with an ultrasound-based real-time tracking system. Their results demonstrated increased accuracy of stone targeting with less shockwaves applied. Smith et al. [32] also compared the stone free rates between the USa localization technique and the traditional FS localization technique. The results revealed equivalent outcomes using the two different stone localization modalities but with the added benefit of no ionization exposure when using the USa technique. A randomized prospective study conducted by Van Besien et al. [33] demonstrated similar results, with the USa SWL stone-free rate not being inferior to the FS SWL, but with no need for ionizing radiation. This is probably most beneficial in pediatric patients, as children are 2–7 times more radiation-sensitive than adults [34]. In a study evaluating the outcomes and radiation exposure of children with cystine stones, Goren et al. [35] demonstrated that USa SWL was more effective and applied less ionizing radiation doses for pediatric patients. Also, in faintly radiopaque stones, such as cystine stones, USa-guided SWL has better visualization of the stones than a fluoroscope.

In the current study, the USa group had a larger baseline stone size. Despite this, the USa group had a significantly better stone-free rate, stone-disintegration rate and a lower retreatment rate; this was regardless of stone size stratification. This result indicates better stone localization and targeting with the assistance of real-time ultrasound, which causes better stone fragmentation and secondarily leads to better stone clearance and lower retreatment rates. As for safety, the USa group had a significantly lower complication rate for smaller stones, and a lower complication rate for larger stones, although this did not reach statistical significance. These results further indicate the theoretical benefit of ultrasound tracking, that is, better accuracy leads to less surrounding tissue damage, which would be more significant in smaller targets.

Previously reported stone-free rate of ESWL ranged around 47 to 92% [2–7]. In our study, the main reason of our relative lower stone-free rates is because that stone-free status was more strictly defined. Stone-free status was defined as the absence of radiographic residual stones in the follow-up imaging, any residual fragments visible on KUB was considered as non-stone-free, even the fragments were less than 2 mm or 4 mm, which was the most commonly used criteria in clinical practice and other studies.

Previous studies had certain limitations, such as small patient numbers or a lack of head to head comparisons [19, 32]. Despite its randomized prospective study design, Van Besein’s study failed to show statistical significance between the different localization methods, this may be related to the small number of studied patients. A strength of the current study compared to others is that we have a large number of study patients, two similar energy source lithotripters using the same SWL protocol, and all procedures were performed by the same two experienced technicians, which provides strong head-to-head comparison while minimizing bias between different institutions and operators.

However, there were several limitations to the present study that need to be addressed. First, its retrospective study design limited the bias control, such as stone composition and anatomic parameters. Second, radiation exposure was not recorded besides self-reported preference by the two technicians. Third, CT was not routinely in every patient to assess factors that affect ESWL results, such as CT values, stone-to-skin distance and BMI. In addition, we compared two different electromagnetic lithotripters. Different lithotripters' acoustic output, efficiency, and collateral effects vary between models. Therefore, further strictly controlled studies are needed to support the conclusion. Lastly, SWL and localization techniques are very operator-dependent. The current study was conducted in one single center and only two technicians operated the machine, thus minimizing inter-operator and institutional bias. Further studies could be conducted in a larger set of patients who are prospectively randomized.

## Conclusions

SWL is a safe and non-invasive way of treating renal stones. this study compared two electromagnetic shock wave machines with different stone tracking systems. LiteMed LM-9200 ELMA lithotripter, which combined ultrasound and fluoroscopic stone targeting outperformed Medispec EM-1000 lithotripter, which used fluoroscopy for stone localization and tracking, with better stone-free rates and disintegration rates, as well as lower retreatment rates and complications with possible reduced radiation exposure.

## Data Availability

Records and data pertaining to this study are in the patient’s secure medical records in Mackay Memorial Hospital and are available from the corresponding author on reasonable request.

## Abbreviations

CT: Computed tomography
SWL: Shock wave lithotripsy
KUB: Kidneys, ureter and bladder
IVU: Intravenous urography
URSL: Ureteroscopic lithotripsy
RIRS: Retrograde intrarenal surgery
PCNL: Percutaneous nephrolithotomy
